# Germline and somatic mtDNA mutations in mouse aging

**DOI:** 10.1371/journal.pone.0201304

**Published:** 2018-07-24

**Authors:** Hong Ma, Yeonmi Lee, Tomonari Hayama, Crystal Van Dyken, Nuria Marti-Gutierrez, Ying Li, Riffat Ahmed, Amy Koski, Eunju Kang, Hayley Darby, Thanasup Gonmanee, Younjung Park, Don P. Wolf, Chong Jai Kim, Shoukhrat Mitalipov

**Affiliations:** 1 Center for Embryonic Cell and Gene Therapy, Oregon Health & Science University, Portland, Oregon, United States of America; 2 Division of Reproductive & Developmental Sciences, Oregon National Primate Research Center, Oregon Health & Science University, Beaverton, Oregon, United States of America; 3 Stem Cell Center, ASAN Institute for Life Sciences, ASAN Medical Center, Seoul, South Korea; University of Cologne, GERMANY

## Abstract

The accumulation of acquired mitochondrial genome (mtDNA) mutations with aging in somatic cells has been implicated in mitochondrial dysfunction and linked to age-onset diseases in humans. Here, we asked if somatic mtDNA mutations are also associated with aging in the mouse. MtDNA integrity in multiple organs and tissues in young and old (2–34 months) wild type (wt) mice was investigated by whole genome sequencing. Remarkably, no acquired somatic mutations were detected in tested tissues. However, we identified several non-synonymous germline mtDNA variants whose heteroplasmy levels (ratio of normal to mutant mtDNA) increased significantly with aging suggesting clonal expansion of inherited mtDNA mutations. *Polg* mutator mice, a model for premature aging, exhibited both germline and somatic mtDNA mutations whose numbers and heteroplasmy levels increased significantly with age implicating involvement in premature aging. Our results suggest that, in contrast to humans, acquired somatic mtDNA mutations do not accompany the aging process in wt mice.

## Introduction

Maternally inherited mammalian mtDNA is typically present at a thousand genomes per cell compared to only two copies for most nuclear genes [1]. MtDNA encodes 13 proteins essential for the mitochondrial respiratory chain along with 22 tRNA and 2 rRNA genes required for intra-mitochondrial synthesis of these proteins [2]. Structural defects in mtDNA can impinge on a wide spectrum of cellular functions and can appear in all copies of the genome (homoplasmy) or coexist with wt mtDNA (heteroplasmy). In the latter case, the percentage of mutant mtDNA must reach a certain minimum threshold before the resulting bioenergetic deficiency can affect cellular function and disease symptoms become evident. A combination of close proximity to reactive oxygen species, lack of protective histones and relatively low genome repair fidelity has been associated with age-related accumulation of somatic mtDNA mutations (i.e. point mutations and large rearrangements or deletions) and subsequently to mitochondrial dysfunction, degenerative diseases and aging itself [3–12]. In addition to somatic mutations, germline mtDNA mutations can be inherited from oocytes and become dispersed during development throughout the body in all or most organs and tissues. Mutations may also arise during early embryonic development and, depending on the affected progenitor lineages, become confined to specific organs or tissues of the body. In contrast, late-onset somatic mtDNA mutations could persist in a mosaic pattern in a few or just within a single cell [13]. While both clonal expansion of preexisting germline or early embryonic mutations [14–20] and the time-dependent accumulation of *de novo* somatic mtDNA mutations [21–23] have been implicated, their interplay and relative impact on aging remains unclear.

The mouse is a convenient model for investigating molecular mechanisms underlying mammalian aging. In particular, *Polg* mutator mice carrying a nuclear gene defect in the proofreading function of the catalytic subunit of mitochondrial DNA polymerase γ, have been critical to the successful assessment of mtDNA mutations [24, 25]. *Polg* mice accumulate high numbers of random mtDNA point mutations during development and display premature aging-like phenotypes, such as weight loss, reduced subcutaneous fat, alopecia, osteoporosis and hearing loss [24, 25]. While both homozygous and heterozygous *Polg* induce somatic mtDNA mutations, only homozygous animals display premature aging [21]; the life span of heterozygous *Polg* mice is relatively normal [26]. These and other observations argue against the premise that acquired somatic mtDNA mutations are solely responsible for premature aging [23]. In addition, the prevalence of somatic mtDNA mutations in wild type (wt) mice and their implication in normal aging remains uncertain [26–28]. Based on analysis of the non-coding D-loop region, the frequency of mtDNA mutations is higher in aged mice than in younger controls [28]. However, it should be noted that mutations in the non-coding region of mtDNA are not necessarily reflective of the entire genome, and there are indications of a disparity in the mutation estimates in coding and non-coding mtDNA regions [29]. In Khaidakov et al., 2003, the presence of mtDNA point mutations was reported in aging mice, however, the origin of these defects was not defined [16].

In an effort to resolve these inconsistencies and define the relative contribution of germline vs. somatic mtDNA defects to the aging process, we examined multiple tissues in wt and *Polg* mice by whole mtDNA sequencing. We show that wt mice carry primaryly germline or early embryonic, but not somatic mutations. We also note that heteroplasmy levels of germline mutations increase with age in wt mice. In addition, a significant increase in heteroplasmy levels of germline and somatic mtDNA mutations was correlated with premature aging in *Polg* mice. These results suggest an important role of germline mtDNA mutations on aging and define similarities and differences between wt and *Polg* mice.

## Results

### Germline, embryonic and somatic mtDNA mutations in wild type mice

**Fig 1:**
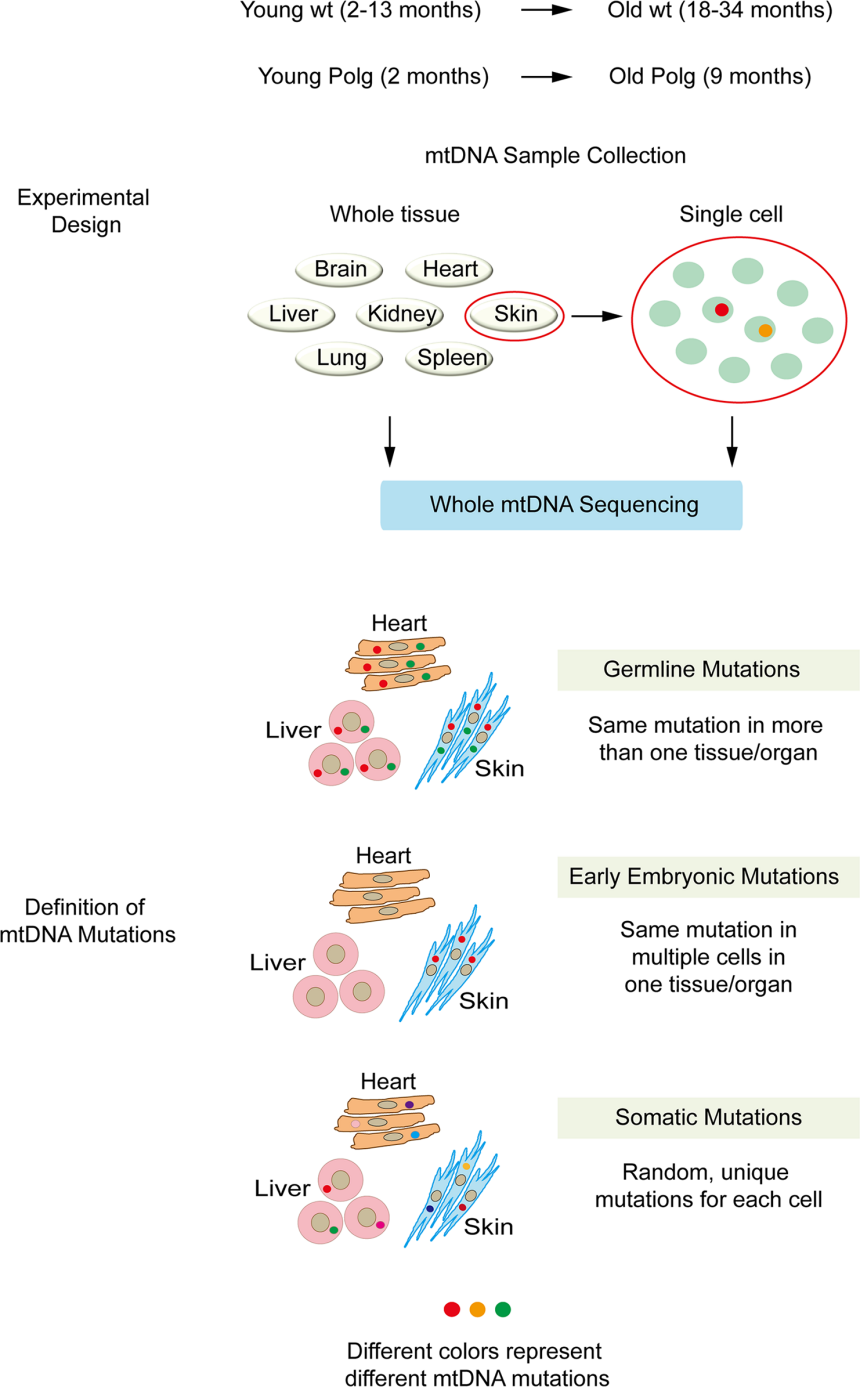
Schematic chart of experimental design. Diagram outlining the experimental groups employed to study aging in the mouse and definition of mtDNA mutations into germline, embryonic and somatic origin. MtDNA samples were collected from bulk tissues or individual skin fibroblast-derived iPSC clones or clonally expanded skin fibroblasts from wt mice (2–34 months) and *Polg* mutator mice (2–9 months). All mtDNA samples were subjected to whole mtDNA sequencing by Miseq (Illumina).

To elucidate the origin of mtDNA mutations and determine their relative role in aging, we examined skin, brain, heart, kidney, liver, lung, and spleen collected from young (2–13 months) and old (18–34 months) wt BDF mice, which were generated by crossing C57BL/6 females with DBA males (Charles River laboratory), and contain the C57BL/6 mitochondrial background (Fig 1). Miseq-based whole mtDNA sequencing (Illumina) was carried out and mtDNA mutations detected at the heteroplasmy level ≥ 2% were called and divided subsequently into germline, early embryonic or late somatic origins (Fig 1). We also categorized mtDNA mutations leading to alterations in the protein-coding sequence as non-synonymous, silent substitutions as synonymous, mutations in RNA-coding genes as RNA mutations, and mutations in the non-coding control region of mtDNA as NCR mutations.

Among five young wt mice examined, four did not have any detectable mutations in either tested tissues or organs (S1 Table). One mouse (10 months) in this group showed two heteroplasmic mutations in skin but absent in brain, heart, kidney, liver, lung or spleen samples. We speculate that these mutations likely impact protein synthesis or mitochondrial complex I activity as one affected an rRNA gene (mt2080G>A) with 95% heteroplasmy and the other was non-synonymous (mt3366G>A) in the ND1 gene (*mt-Nd1*) at 25% heteroplasmy. We reasoned that late acquired somatic mtDNA mutations could be present in individual cells, with each cell harboring a unique mutation not shared with other cells. Thus, screening bulk tissues containing mtDNA from a million cells may not be sensitive enough to reveal somatic mtDNA mutations in individual cells [13]. Therefore, we established primary cultures of skin fibroblasts (SF), sub-cloned single fibroblast progenies and analyzed individually a minimum of 10 clones per mouse. In selected mice, we also derived 10 iPSC clones from each SF sample to increase sample size and analyzed mutations in each individual iPSC line (S2 Table). Again, no mutations were detected in individual SF or iPSC clones from the four young wt mice, whereas, we confirmed 2 mutations (2080 G>A and 3366 G>A), found in the bulk skin sample from the fifth animal (10 month old male in S1 Table), in individual SF and iPSC clones. We also detected an additional mutation (15264 G>A) in two SF clones from this animal (S1 and S2 Tables). As expected, heteroplasmy levels varied among different clones (S2 Table). Since variants were shared among individual skin cells, these mutations probably occurred during early embryonic development in skin progenitor cells and, thus, were of early embryonic origin (S1 Table) [30]. However, we cannot exclude the possibility that these mutations were inherited but selectively distributed to some tissues.

In the old wt group, six of eight mice carried mtDNA mutations, while the remaining two were free of any detectable mutations in all tested tissues (S1 Table). Among a total of 13 mutations, seven were present only in skin or spleen, suggesting an early embryonic origin. The remaining six mutations were present in multiple organs of the same animal (S1 Table) and identified as germline origin, since inherited mutations originating from an oocyte are likely to be distributed (shared) to most or all organs and tissues of the animal [31]. Detailed analysis showed that 11/13 (85%) of these variants were non-synonymous point mutations or insertion/deletions in protein-coding or RNA-coding genes (S1 Table). All mutations with heteroplasmy levels >8% were confirmed by Sanger sequencing.

We also screened individual SF and iPSC clones from wt old mice and corroborated the presence of embryonic origin skin mutations in either multiple or all clones (S1 and S2 Tables). In addition, we detected two new early embryonic mutations in multiple SF clones from the two wt old mice (31 and 34 month old males in S2 Table), but not in iPS cells from same SF, suggesting that some mutations could be lost during reprogramming through a genetic bottleneck [32]. However, no somatic mutations were detected in either individual SF or iPSC clones suggesting that wt mice predominantly carry either germline or early embryonic mtDNA mutations. Unlike humans [13] late acquired somatic mtDNA mutations were not detected in mouse skin tissues suggesting different genetic mechanisms of aging.

### Analysis of mtDNA mutations in young vs. old wild type mice

**Fig 2:**
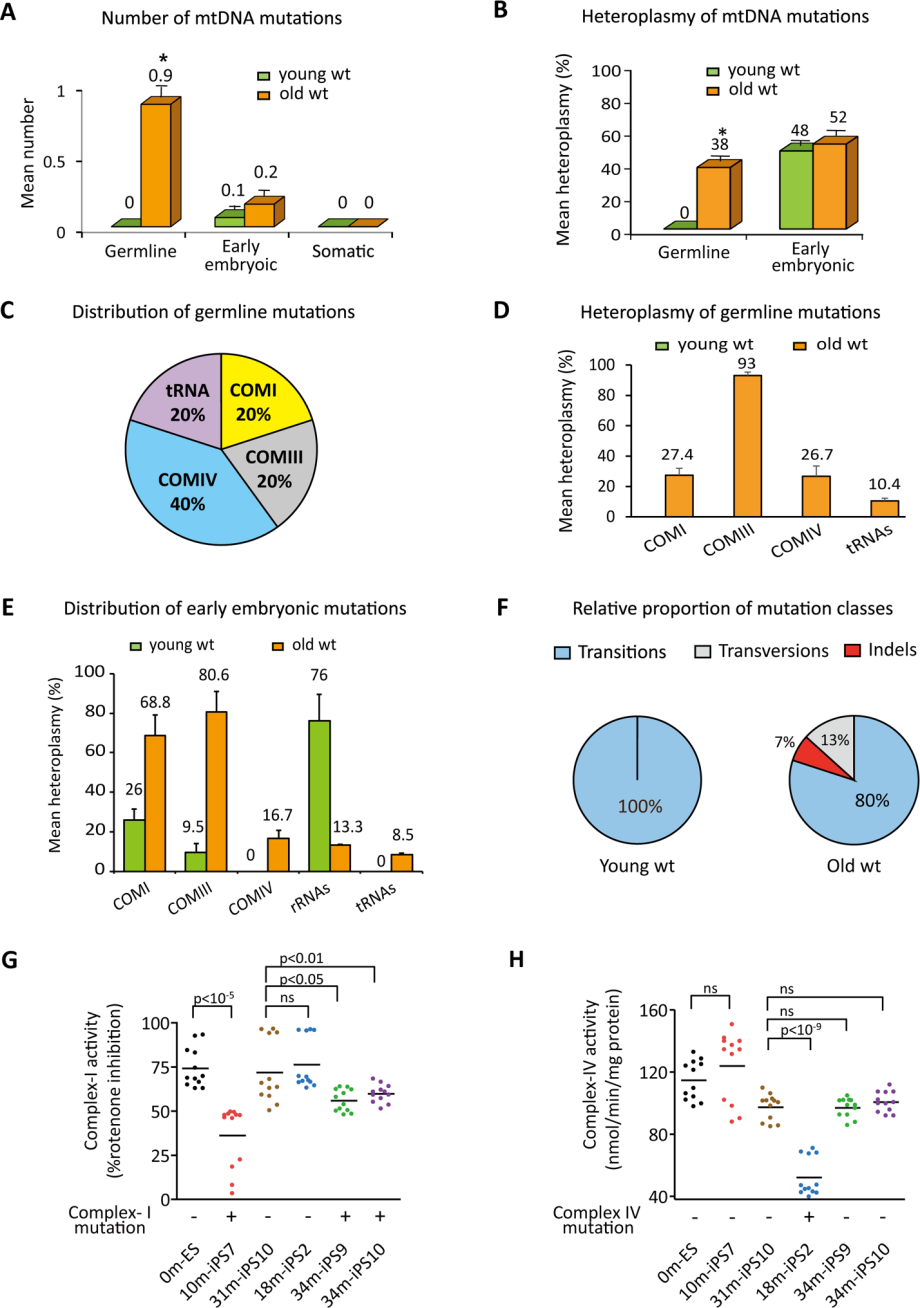
Characterization of mtDNA mutations in wild type mice with aging. (A) Quantification of mtDNA mutations (mean ± SEM; asterisk, P < 0.05, Student’s t-test) for different mutation types in young wt mice at age 2–13 months (green bars; n = 31) and old mice at age 18–34 months (orange bars; n = 44). Somatic mutations were undetectable in wt mice. Asterisk represents a significant increase in number of germline mutations with age. (B) Mean heteroplasmy levels of non-synonymous germline and early embryonic mutations as a function of age. Error bars, mean ± SEM. Asterisk represents a significant increase in the mean heteroplasmy levels of non-synonymous germline mutations in old wt mice compared to the young group (P < 0.05, Student’s t-test). (C) Pie chart showing gene distribution of non-synonymous germline mutations in protein-coding and RNA coding genes in old wt mice. (D) Bar graphs showing mean heteroplasmy levels for non-synonymous germline mutations in protein-coding and RNA-coding genes in old wt mice. (E) Heteroplasmy of non-synonymous mutations in protein-coding and RNA-coding genes of early embryonic origin. (F) Pie chart showing relative proportion of mutation types in young and old wt mice. (G) Mitochondrial OXPHOS complex I activity in iPS cells carrying non-synonymous mutations in protein-coding genes and in age-matched control ESCs or iPS cells. The complex I activity was measured in cell homogenates (n = 12 per cell line, technical replicates) and was expressed as “% rotenone inhibition”. 10m-iPS7, 34m-iPS9 and 34m-iPS10 cells displayed reduced activities compared to controls. (H) Mitochondrial OXPHOS complex IV activity in iPS cells carrying non-synonymous mutations in protein-coding genes and in age-matched control ES or iPS cells. The complex IV activity was measured in cell homogenates (n = 12 per cell line, technical replicates) and was expressed as “nmol / min / mg protein”. 18m-iPS2 cells showed decreased activity compared to controls. In (G and H), ns denotes p ≥ 0.05.

To further define age-related changes in mtDNA stability, we compared the average number and heteroplasmy levels of each mutation type in wt mice. On average 0.9±0.1germline mutations per tissue were detected in old wt mice while no germline variants were found in young animals (P < 0.05, Student’s t-test, Fig 2A and Table 1). In this case, mutations that did exist must have been undetectable due to low heteroplasmy levels (below the 2% detection level) while subsequent clonal propagation during aging accounts for the heteroplasmy increase. Indeed, five non-synonymous or RNA-coding germline mutations found in old wt mice were present at heteroplasmy levels ranging between 5 and 100% with an average of 38%±5.9 (S1 Table, red fonts; Fig 2B, P < 0.05, Student’s t-test). However, there was no difference in the mean number of early embryonic mutations in old when compared to young mice (Fig 2A and 2B; Table 1). In addition, all 3 embryonic mutations detected in young mice and 8 out of 9 found in old mice were non-synonymous or RNA coding mutations, (S1 Table, blue fonts), but no significant increase in the heteroplasmy level with aging was seen (Fig 2B and 2E).

**Table 1:** Summary of mtDNA mutations detected in wild type and *Polg* mice.

Groups	Age (month)	Total No. of mice	No. of mtDNA mutations (mean)		
			Germline	Early embryonic^c^	Somatic^d^
Young wt	2–13	5	0±0^a^	0.1±0.1	0
Old wt	18–34	8	0.9±0.1^a^	0.2±0.1	0
Young *Polg*	2	3	0.3±0.2^b^	8.4±3.7	59.2±7.0^d^
Old *Polg*	9	3	4.3±0.8^b^	5.8±2.4	95.4±10.9^d^

^a^ The mean numbers of germline mutations were calculated as mean/tissue and were significantly different between young wt and old wt (P < 0.05, Student’s t-test). n = 31 for young wt; n = 44 for old wt.

^b^The mean numbers of germline mutations were significantly different between young *Polg* and old *Polg* (P < 0.05, Student’s t-test); n = 8 for young *Polg*; n = 14 for old *Polg*.

^c^The mean numbers of early embryonic mutations were calculated as mean/tissue or single SF clone; n = 31 for young wt; n = 44 for old wt; n = 14 for young *Polg*; n = 20 for old *Polg*.

^d^The mean numbers of somatic mutations were calculated as mean/iPS cell or single SF clone and were significantly different between young *Polg* and old *Polg* (P < 0.05, Student’s t-test); n = 40 for young wt; n = 70 for old wt; n = 10 for young and old *Polg*.

We next looked at the distribution of germline mutations in old mice. Protein-coding gene mutations were grouped according to the OXPHOS enzyme complexes to which their products belonged. Among all germline mutations detected, 40% resided in the genes of mitochondrial complex IV (COMIV) while the remaining 60% were evenly distributed between complex I (COMI), complex III (COMIII) and tRNAs (Fig 2C). COMIII mutations were present at higher heteroplasmy levels (93%±2.3) compared to tRNA mutations (10%±1.8). COMI and COMIV gene mutations were present at intermediate heteroplasmy levels (Fig 2D and S1 Table).

Oxidative damage caused by reactive oxygen species (ROS) has long been considered a primary driver of mtDNA mutations [33]. Particularly, G>T/C>A transversion mutations are thought to result from oxidative mtDNA damage [34]. Therefore, we carefully inspected frequency of transition, transversion or indel mutations in wt mice. In both young and old mice, mtDNA mutation types were biased towards transitions. All mutations (3/3) found in young mice and 12 out of 15 (80%) mutations in old animals were transition mutations with G>A or T>C as dominant types (S1 Table and Fig 2F). The remaining 3 mutations in the old mice included 1 G>T transversion and 2 indels (Fig 2F and S1 Table). These results suggest that the frequency of each mutation type is similar between young and old mice without noticeable increase in G>T transversions (Fig 2F). These observations are in agreement with previous studies [35, 36] supporting the view that oxidative damage is not the major driver of mtDNA mutagenesis.

### Mitochondrial OXPHOS in iPSCs carrying mtDNA mutations

Mitochondrial OXPHOS complex activities are essential for mitochondrial function and OXPHOS dysfunctions are linked to a variety of human disorders including primary mitochondrial diseases caused by mutations both in the mitochondrial and nuclear DNA [37] as well as in aging [38]. Non-synonymous substitutions or indel mutations may alter coding of amino acids, which in turn, can result in changes of protein function. The majority (67%, 12/18) of mtDNA mutations detected in wt young and old mice were non-synonymous substitutions or indels. To further invest the potential impact of those mutations on mitochondrial respiratory function, we measured OXPHOS complex activities in undifferentiated iPS cells carrying non-synonymous mutations in complexes I or IV (S2 Table). Enzymatic activity of complex I and IV were assayed spectrophotometrically in iPS clone7 (10m-iPS7), clone2 (18m-iPS2), clone9 and clone10 (34m-iPS9 and 34m-iPS10) derived from 10 month male, 18 month female and 34 month male (S2 Table), respectively. Age-matched iPS cells with no mutations (31m-iPS10) (S2 Table) and ES cells derived from wt embryos (0m-ES) were included as controls. As expected, iPS cells carrying mutation in complex I, i.e. 10m-iPS7 with 96% heteroplasmy for 3366 G>A *mt-Nd1* mutation and 34m-iPS9 and 34m-iPS10 with homoplasmy for 13029 G>A *mt-Nd5* mutation, displayed significantly reduced complex I activity (36.1%±2.3 in 10m-iPS7, 55.9%±1.8 in 34m-iPS9 and 59.8%±1.5 in 34m-iPS10), when compared to controls (0m-ES, 74.2%±3.2 and 31m-iPS10, 72.1%±5.2). As expected, 18m-iPS2 cells carrying mutation in complex IV presented normal complex I activity (76.3%±4.2) similar to controls (Fig 2G). In contrast, 18m-iPS2 cells displayed significantly reduced complex IV activity (52.1±3.6 nmol/min/mg protein) while all other iPS cells displayed normal complex IV activities (from 97±1.8 to 123.9±6.5 nmol/min/mg protein) similar to controls (97.3±2.4 nmol/min/mg protein in 31m-iPS10 and 114.7±3.7 nmol/min/mg protein in 0m-ES) (Fig 2H). These results suggest functional implications of specific mtDNA variants observed in mice (Fig 2G and 2H).

In summary, late-onset somatic mutations were undetectable in wt mice, even at the single cell level, suggesting species specific differences from humans. However, germline mutations detected in old but not in young mice implied that heteroplasmy levels increased gradually with advancing age (Table 2). Most of these mutations were non-synonymous substitutions and presented at high heteroplasmy levels likely affecting metabolic function in aged tissues and organs.

**Table 2:** Dynamics of mtDNA mutations during mouse aging.

	Germline mutations	Early embryonic mutations	Late onset somatic mutations
Origin	In oocytes	During early embryonic development	During postnatal development
Number of mutations in wt mice	Significantly increased with age	No change with age	Undetectable
Heteroplasmy changes in wt mice	Significantly increased with age	No change with age	Undetectable
Number of mutations in *Polg* mice	Significantly increased with age	No change with age	Significantly increased with age
Heteroplasmy changes in *Polg* mice	Significantly increased with age	No change with age	Significantly increased with age

### Clonal propagation of germline mutations during *Polg* aging

*Polg* mice show increased loads of mtDNA mutations linked to reduced lifespan and premature onset of aging-related phenotypes [23–25]. We examined changes in the number and heteroplasmy of mtDNA defects during aging in homozygous *Polg* mice from 2 to 9 months of age (n = 2). We initially biopsied skin tissue from young living 2 month old mice (young *Polg*) and then analyzed skin again, along with brain, heart, kidney, liver, lung and spleen in the same animals after sacrifice at 9 month of age (old *Polg*). Germline, early embryonic and late onset somatic mtDNA mutation types were called based on the criteria used for wt mice.

**Fig 3:**
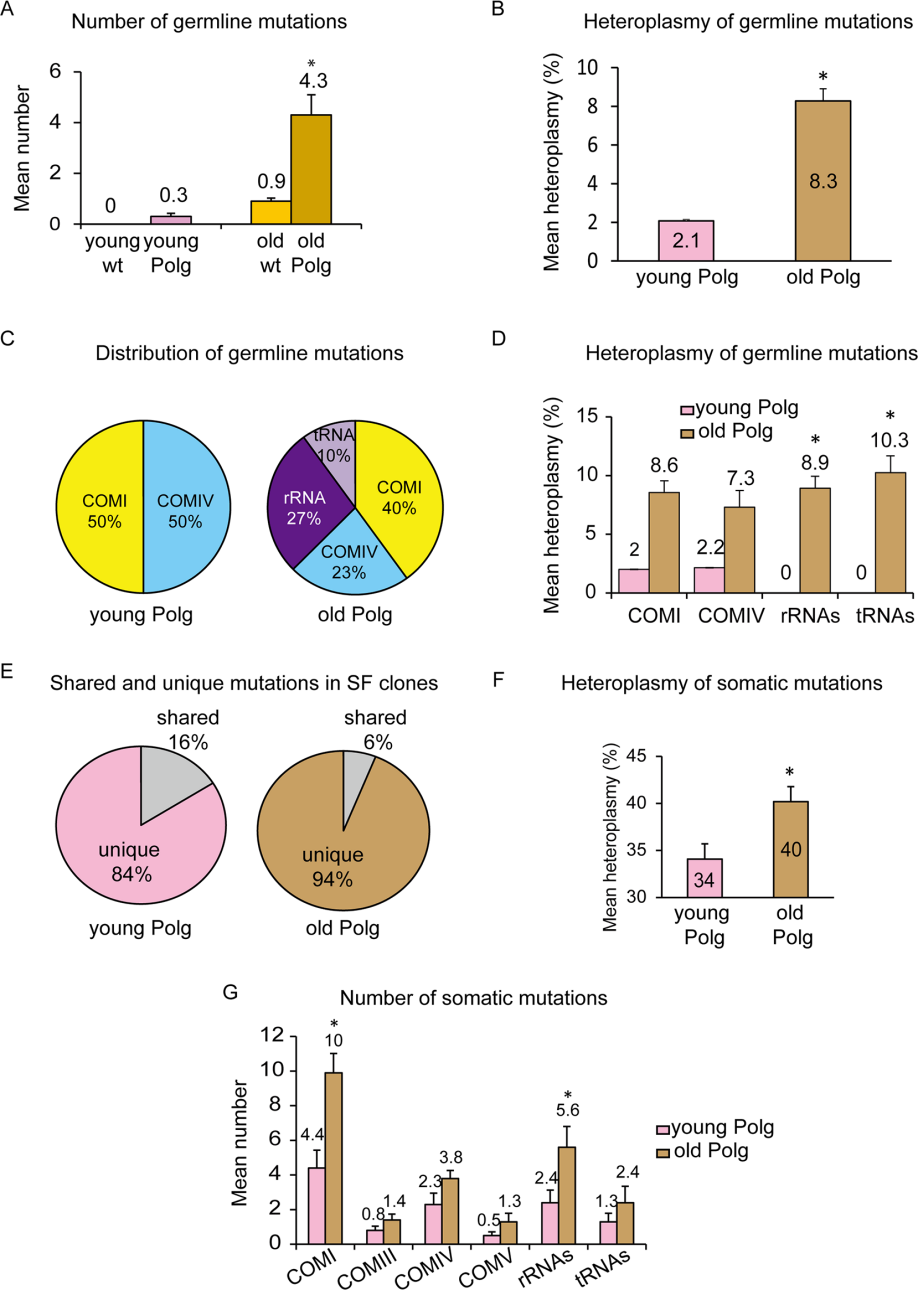
Characterization of mtDNA mutations in homozygous *Polg* mice with aging. (A). Comparison of mean number of germline mutations in wt and *Polg* mice at young and old age (n = 31 for young wt, n = 8 for young *Polg*, n = 44 for old wt and n = 14 for old *Polg*). Error bars, mean ± SEM. Asterisk indicates a significant increase in the number of mutations per tissue in old *Polg* compared to the old wt (P < 0.05, Student’s t-test). (B). Mean heteroplasmy levels of non-synonymous germline mutations with ≥2% heteroplasmy in *Polg* mice (mean ± SEM; asterisk, P < 0.05, Student’s t-test). (C) Pie charts showing gene distributions of non-synonymous germline mutations in young *Polg* mice (2 months, left) and old *Polg* mice (9 months, right). (D) Bar graphs representing the mean heteroplasmy levels of non-synonymous germline mutations in protein-coding and RNA-coding genes in *Polg* mice (asterisks, P < 0.05, Student’s t-test). (E) Pie charts showing the distribution of shared and unique mtDNA mutations detected in single skin fibroblast (SF) clones in young and old *Polg* mice. (F) Mean heteroplasmy changes for non-synonymous somatic mutations with ≥15% heteroplasmy in *Polg* mice. Error bars, mean ± SEM. Asterisk, P < 0.05, Student’s t-test. (G) Changes in number of non-synonymous somatic mutations with heteroplasmy levels ≥ 15% among different gene types with *Polg* mice aging. Error bars, mean ± SEM. Asterisks, P < 0.05, Student’s t-test.

In contrast to wt mice, germline mutations were already detectable in skin tissue of young *Polg* mice and in multiple tissues of old *Polg* animals, their numbers increasing significantly with age (from 0.3±0.2 to 4.3±0.8, P < 0.05, Student’s t-test) (Fig 3A, Table 1 and S3 Table). There were no age-related changes in the number of early embryonic mutations (Table 1).

**Fig 4:**
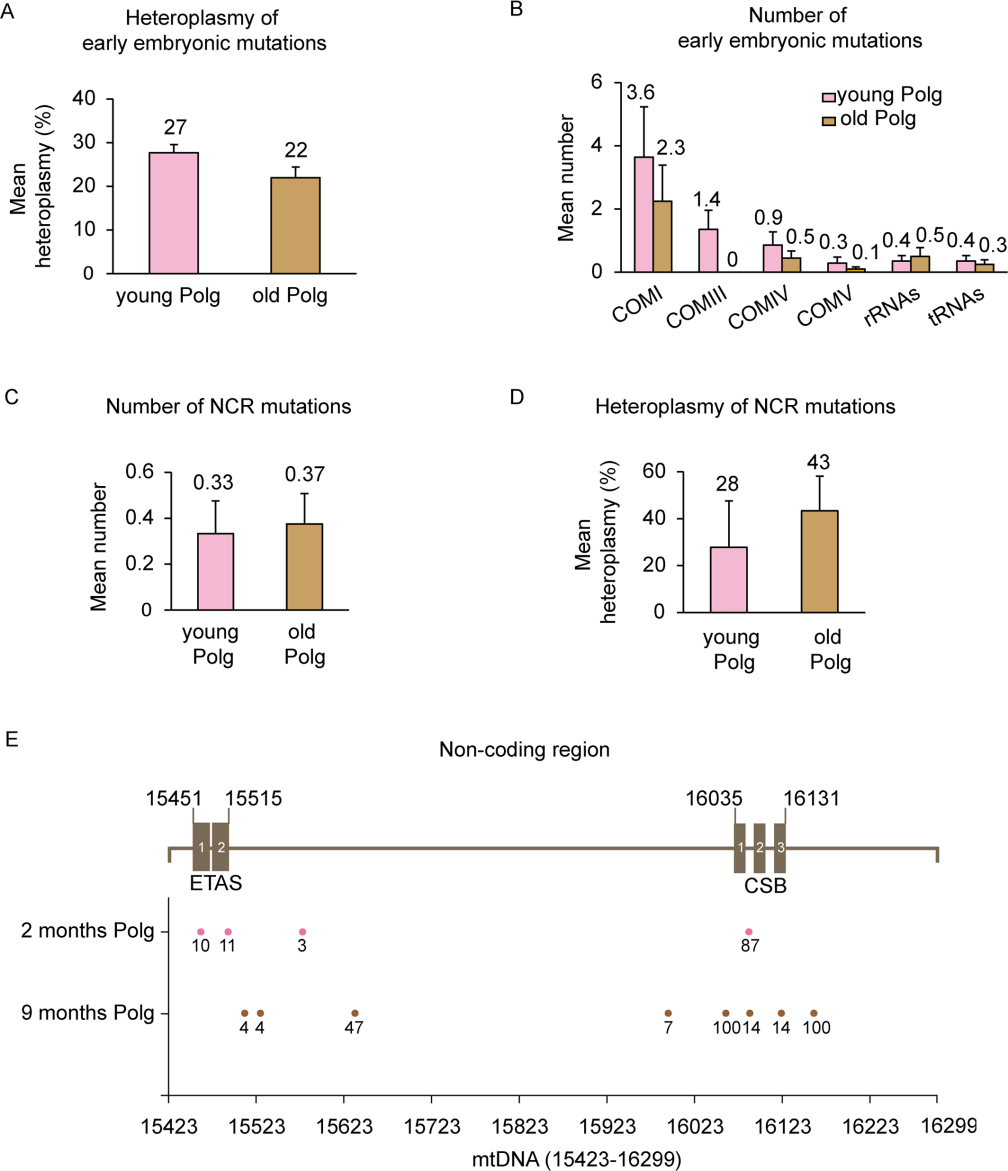
Early embryonic and NCR mtDNA mutations in *Polg* mice. (A) Bar graphs representing changes in mean heteroplasmy of early embryonic mutations with *Polg* mice aging. Error Bars, mean ± SEM. (B) Distribution of non-synonymous early embryonic mtDNA mutations among different genes. Error bars, mean ± SEM. (C) Quantification of mtDNA mutations in the non-coding region (NCR) in *Polg* mice (n = 12 for 2 months; n = 24 for 9 months). Error bars, mean ± SEM. (D) Bar graphs representing mean heteroplasmy levels of NCR mtDNA mutations in *Polg* mice with aging. Error bars, mean ± SEM. (E) Summary of mtDNA mutations found in the NCR region (mtDNA15423-16299) in *Polg* mice. Dots represent mtDNA mutations and numbers under dots represent the heteroplasmy levels. ETAS indicates the extended termination associated sequence and CSB indicates the conserved sequence block.

We also compared changes in mean heteroplasmy of non-synonymous germline and early embryonic mutations in *Polg* mice during aging. While there were no changes in early embryonic mutations (Fig 4A, S4 Table), those of germline origin increased significantly in the old *Polg* group compared to the same animals at a younger age (from 2.1±0.1 to 8.3±0.6, P < 0.05, Student’s t-test) (Fig 3B).

Similar to wt mice, the majority (65%, 11/17) of germline mutations in *Polg* mice were non-synonymous (S3 Table). Those detected in young *Polg* mice were predominantly in protein-coding genes with equal distribution between the COMI and COMIV genes. In old *Polg* mice, 27% were in rRNA genes, 10% in tRNA and 63% in protein-coding genes (Fig 3C). Germline mutations in rRNA and tRNA genes were undetectable in young *Polg* but became evident in several organs of the same animals at 9 months (P < 0.05, Student’s t-test, Fig 3D and S3 Table), indicating that these mutations are clonally expanded with aging. The distribution of non-synonymous early embryonic mutations did not change with aging of *Polg* mice (Fig 4B and S4 Table).

These findings suggest that clonal expansion of potentially pathogenic germline mutations is a common mechanism that accompanies the aging process in both wt and *Polg* mice (Table 2).

### *De novo* and recurring germline mtDNA mutations in *Polg* mice

Germline mtDNA mutations found in *Polg* female offspring could be recurrent mutations inherited from previous generations or *de novo* mutations arising during oogenesis. To define the germline mutation spectra in *Polg* mice, we crossed two homozygous *Polg* females with wt C57BL/6 males and analyzed mtDNA mutations in multiple tissues/organs of the *Polg* mothers (4 month old) and their offspring (age of 3–12 days).

In the *Polg* family 1, two low (2–3%) heteroplasmy mutations were detected; one in lung (9056 C>T) and another in skin tissues (13053 delC in) of the mother (*Polg*-4). We called these variants as of early embryonic origin. In her 3 offspring, pup1 carried 6 mutations including 5 of germline origin; pup2 had 8 mutations including 4 germline, and pup3 showed only one mutation in the kidney only (S5 Table). No shared mutations between the mother and her offspring or between siblings were found in this *Polg* family indicating that all germline mutations in pups were *de novo* in origin (S5 Table). In the *Polg* family 2, the mother (*Polg*-5) carried three mutations including two of germline origin. Each of her 3 pups carried between 24 and 27 individual mutations and the majority of them were of germline origin (S5 Table). Two germline mutations were shared between the mother and her pups or between siblings suggesting recurring germline mutations. A mt15104C>T variant (blue font in S5 Table) was detected in the mother’s skin (6% heteroplasmy) and in all 7 tissues (12–15% heteroplasmy) of her pup1. The Mt6612C>T mutation (red font in S5 Table) was seen in all tissues of pup2 (13–28% heteroplasmy) and in kidney and lung of pup3 (5–6% heteroplasmy). Most other germline mutations were present in only one animal (S5 Table). These data indicate that the majority of germline mutations in *Polg* offspring are *de novo* with very few recurring preexisting variants.

### Somatic mtDNA mutations in *Polg* mice

Homozygous *Polg* mice develop aging phenotypes prematurely and have significantly shorter lifespans (approximately 3-fold) compared to their wt counterparts and it is believed that somatic mtDNA mutations acquired later in life play a major role in this phenomenon [23, 24]. We screened multiple, randomly-selected SF clones (n = 10) for each *Polg* mouse at 2 months of age and compared the outcome with the same animal sampled at 9 months. As expected, by 2 months of age, SF clones had already acquired substantial numbers of mtDNA mutations. A total of 705 mutations was discovered in 10 SF clones, 113 (16%) of which were shared either with other clones or also detected in other tissues from the same animal (S6 Table). The remaining 592 mutations (84%) were clonally unique seen only in one SF clone from the same animal and thus were categorized as somatic mutations (Fig 3E and S6 Table). In old *Polg* mice, a total of 1016 mutations was found in 10 SF clones. Among those, 62 (6%) were shared with other clones as either germline or early embryonic mutations. The remaining 954 mutations (94%) were unique, found in only one clone as somatic mutations (Fig 3E and S6 Table).

The mean number of somatic mtDNA mutations (59.2±7.0) detected per SF clone in young *Polg* mice increased significantly during aging (95.4±10.9) (Table 1). To gain further insights into how somatic mtDNA mutations affect premature aging, we examined changes in their pathogenicity and heteroplasmy levels. Mean heteroplasmy levels of somatic mutations in non-synonymous protein and RNA-coding genes increased significantly with *Polg* age (from 34±1.6% to 40±1.6%; P < 0.05, Student’s t-test, Fig 3F). Similarly, the mean number of mutations in rRNA-coding genes increased significantly in old *Polg* (from 2.4±0.7 to 5.6±1.2, P < 0.05, Student’s t-test, Fig 3G). All 24 rRNA mutations found in young *Polg* mice were at relatively low heteroplasmy levels (≤80%), while of the 56 found in old *Polg*, eight (14%) were homoplasmic or at ≥80% (S7 Table, red font). In addition, the mean number of somatic mutations in COMI genes increased significantly in old *Polg* (from 4.4±1.0 to 10±1.1, P < 0.05, Student’s t-test, Fig 3G). Among 99 COMI mutations found in old *Polg*, 11 were at heteroplasmy ≥80%. In contrast, all 44 COMI mutations in young *Polg* mice were at ≤80% (S7 Table). Mean numbers of somatic mutations in COMIII, COMIV, COMV, and tRNA-coding genes did not change with age (Fig 3G).

In summary, similar to wt mice, *Polg* animals display increase in heteroplasmy levels of non-synonymous, germline mtDNA mutations with age. Thus, clonal propagation leading to increased heteroplasmy levels appears to be a common phenomenon in mouse aging. In addition, *Polg* mice show a substantial number of somatic mtDNA mutations whose mean number and heteroplasmy levels also increased significantly with aging. Since the number of somatic mutations in COMI and rRNA genes significantly increase with *Polg* aging, likely affecting OXPHOS function and protein synthesis, they could be implicated in driving mitochondrial dysfunction during premature aging (Table 2).

### Mutations in the non-coding control region of mtDNA

The major non-coding control region (NCR) of mtDNA, including the D-loop area, is important for regulating mtDNA replication and transcription [39, 40]. Previous studies show that both mtDNA replication and transcription are impaired in aged tissues [15, 41]. Interestingly, mutations in the NCR region are rarely reported even in *Polg* mice [27, 42]. Consequently, we screened both wt and *Polg* mice. As expected, wt mice did not carry any detectable NCR mutations even at advanced age. In contrast, mutations in NCR were found in *Polg* mice but their numbers were low compared to mutations in coding regions. Furthermore, the number and heteroplasmy levels of NCR mutations did not change with age (Fig 4C and 4D). A total of 12 mutations was found in this region including 4 somatic mutations (heteroplasmy 10–87%) in young *Polg* with 6 somatic and 2 early embryonic mutations in old *Polg* mice (heteroplasmy 4–100%). Two mutations (heteroplasmy 10–11%) in young *Polg* were confined to the extended termination associated sequence region (ETAS) (Fig 4E). The ETAS region is highly conserved in mammals but its actual function remains uncertain. One mutation (heteroplasmy 14%) in old *Polg* was found in the conserved sequence blocks (CSB), crucial elements controlling mtDNA replication and transcription [39, 43]. Since NCR is critical for mtDNA replication, it is likely that functional mutations in this region cannot be propagated and will eventually lead to their elimination.

## Discussion

In this study, we evaluated the accumulation dynamics of mtDNA mutations during mouse aging based on their occurrence in single or multiple organs, tissues and cells of the same organism and thus allocation to germline, embryonic or somatic origin. Our results indicate that wt mice do not display any detectable somatic mtDNA mutations, even with advanced age. However, old wt mice carry a higher number of non-synonymous, germline mutations compared to young animals. These mutations likely originated in oocytes since they were found in multiple organs and tissues of the same animal. However, perhaps due to low heteroplasmy levels, many germline mutations remained undetectable at the younger age. The preferential amplification of these rare germline variants could result in substantial increases in heteroplasmy levels during aging. A few early embryonic mutations (common for one tissue or organ) were also detected in wt mice but did not change with age, that is, they might be fixed during early embryogenesis. Possible roles of mtDNA mutations in wt mouse aging have generated debate that may reflect differences in the detection technology employed, the tissues examined and the origin of mtDNA mutations (total mutations vs. somatic mutations) [26–28]. The present study, strictly defining germline, embryonic and acquired mutations, provides insights into the relative contribution of each to mouse aging.

The majority of mtDNA mutations detected in wt mice were non-synonymous substitutions or indels and resulted in reduced mitochondrial OXPHOS complex I and IV activities *in vitro* in mutation-carrying iPS cells. Mitochondrial dysfunction is a hallmark of aging, leading to a loss of cellular homeostasis and organismal health [44, 45], but the mechanisms underlying the relationship between mitochondrial dysfunction and the aging process are not well understood. Deficient OXPHOS and loss of ATP for cellular processes could lead to loss of OXPHOS enzymes, particularly Complex I, elevated NADH/NAD^+^ ratio, and reduced levels of NAD^+^ [46]. NAD^+^ is needed for the generation of PARP (Poly (ADP-ribose) polymerase) which, in turn, is critical for the identification of single-strand DNA breaks and the signaling to activate enzymatic DNA repair. Reduced levels of NAD^+^ may also lead to reduced activity of Sirtuin 1, which may further aggravate age-related organ pathology [47]. Further quantitation of non-synonymous mutations in mtDNA with assignments of those implicated in mitochondrial function i.e., respiration deficits *in vivo*, will supports a plausible cause and effect relationship in mtDNA based mitochondrial disease and in the aging process.

In *Polg* mice, both germline and late acquired somatic mutations were evident and their load increased with aging. These observations are in agreement with findings reported by others [24, 25, 48]. In addition, we found age-related increases in the number and heteroplasmy level for somatic mutations in genes coding 12S and 16S ribosome RNAs and oxidative phosphorylation complex I. These mutations, especially at high heteroplasmy, are likely affecting OXPHOS and consequently aggravating aging phenotypes. The involvement of both germline and somatic mutations is consistent with the hypothesis that such a dual effect is likely responsible for premature aging seen in homozygous *Polg* mice [23]. However, clonal amplification of preexisting germline mutations appears to be a universal mechanism causing OXPHOS defects responsible for aging in both wt and *Polg* mice.

In the current study, the heteroplasmy detection threshold was conservatively set at ≥2%, predicated in part, by the observation that heteroplasmy levels >1.6% are likely of biological origin rather than representing technical artifacts [49]. Obviously, we cannot exclude the possibility that very low heteroplasmy (< 2%) somatic mutations exist in bulk tissues of wt mice. We defined late-onset somatic mutations based on their unique existence at the single cell level in SF or iPSC clones. Due to technical limitations of mtDNA mutation analysis in individual, particularly post mitotic cells, the extent of somatic mtDNA mutations in more energy demanding tissues is less well known.

*Polg* mice were also known to carry mtDNA deletions [24, 50]. However, we did not detect such mtDNA defects in this study, possibly due to limitations of whole genome sequencing in identifying low heteroplasmy deletions.

Unlike wt mice, both germline and somatic mutations were observed in humans [13, 51–53]. However, their individual role and involvement during human aging is unknown. Similar to *Polg* mice, low heteroplasmy germline mutations could be clonally amplified with age and potentiate the effects of late-acquired somatic mutations ultimately contributing to age-onset disease [52, 54–56]. Other studies suggested that impact of mtDNA mutations on normal aging may differ significantly between humans and mice [57–59]. Further elucidation of genetic mechanisms underlying aging should yield valuable insights into the role of mtDNA mutations in mammalian aging and disease with the promise of developing clinically valuable approaches to limiting their effects.

## Materials and methods

### Animals

All mice were housed in pathogen-free conditions under controlled lighting conditions (12 h light/12 h dark) in the Small Lab Animal Unit at the Oregon National Primate Research Center. All animal procedures were approved by the Institutional Animal Care and Use Committee (IACUC number IP00000982) at Oregon Health & Science University. Guidelines in the approved protocols were fully followed in the current study.

### Mouse strains

Mouse strain of B6D2F1 (BDF), generated by crossing C57BL/6 females with DBA/2 males (Charles River laboratory), was used in this study as the wild type (wt). BDF contains C57BL/6 mitochondria background. A total of 13 wt mice with age ranging from 2 to 34 months without observed pathogenic phenotype, was analyzed. Homozygous *PolgA*^mut/mut^ (C57BL/6N-*Polg*mut/N) [24] mice carrying C57BL/6 mtDNA were also included in the study. Heterozygous *PolgA*^mut/wild^ were generated by crossing a homozygous *PolgA*^mut/mut^ female with a wild C57BL/6 male.

### Tissue collection

Multiple tissues (brain, heart, kidney, liver, lung, spleen, and ear skin) were collected from wt mice at various ages. For *Polg* mice, ear skin tissue was first collected at 2 months of age, and then brain, heart, kidney, liver, spleen, and ear skin samples were collected from the same mice euthanized at 9 months. Multiple tissues (brain, heart, kidney, liver, lung, spleen, and skin) were also collected from *Polg* mothers and their heterozygous pups to study transmission of *de novo* and recurring germline mtDNA mutations.

### Skin fibroblast isolation, culture and single cell sub-cloning

Skin samples were incubated in 0.1% collagenase (Type IV) for 30 min and then plated into 25-mm flasks containing DMEM/F12 medium supplemented with 10% fetal bovine serum (FBS). Single cells were seeded with limited dilution and subcloned before extracting mtDNA for sequencing.

### Generation of iPSCs from skin fibroblasts

Lentiviral vectors carrying *Oct4*, *Sox2*, *C-myc*, *Klf4* and *RTTA* genes (Addgene) were transfected with pPACKH1 (SBI) and PureFection (SBI) in 293T cells. Supernatants were collected every 24 hours during 3 days starting 24 hours after transfection and filtration through a 0.45 um pore size cellulose acetate filter. Cells (1 x 10^5)^ per well were plated in 6-well culture dishes containing DMEM/F12 medium with 10% FBS one day before transduction. Cultured cells were transduced with filtered supernatants containing viral vector. Three days after transduction, cells were re-plated onto 100-mm dishes containing feeder layers of mitomycin C inactivated mouse embryonic fibroblasts (mEFs) with mouse ES cell media: KODMEM (Invitrogen) supplemented with 20% KSR, 1 mM L-glutamine, 0.1 mM nonessential amino acids (NAA), 0.1 mM β-mercaptoethanol (Sigma), penicillin-streptomycin and 1,000 units/ml LIF (Millipore). Doxycycline was added at a final concentration of 2 μg/ml and medium was changed every day. Colonies appeared 3–4 weeks after transduction and 10 colonies with typical mouse pluripotent stem cell morphology were randomly picked up and propagated for each iPSC line.

### Derivation and culture of ESCs

Superovulated C57BL/6 females were paired with DBA/2 males (Charles River laboratory). Embryos at morula-blastocyst stage were flushed, collected from the uterus of E2.5 females, and cultured in KSOM media for 24 h. The zona pellucida was removed with acidic Tyrode’s solution (Sigma-Aldrich) and blastocysts were individually transferred into mitomycin C treated mEF feeder layers in ES medium: KODMEM (Invitrogen) containing 1mM L-glutamine, 0.1mM β-mercaptoethanol, 0.1mM NAA, 100 units/ml penicillin, 100 μg/ml streptomycin, 1,000 units/ml LIF (Millipore), 10% FBS and 10% KSR (Invitrogen).

After ICM outgrowth, colony was picked up manually and dispersed into single cells using 0.15% trypsin solution (Sigma-Aldrich) before seeding on mEF in ES medium containing 1 μM PD0325901 (Axon) and 3 μM CHIR99021 (Axon).

### DNA extraction and full length mtDNA amplification

DNA was extracted from tissues, skin fibroblasts and iPSCs using a Gentra DNA extraction kit (Qiagen). DNA masses were measured by NanoDrop 2000 spectrophotometry (NanoDrop Technologies). Full length mtDNA was amplified by single PCR reaction for skin fibroblasts and iPSCs using primers, F-3338 CCCCTTCGACCTGACAGAAGGAGAATC and R-3337 GCCCGGTTTGTTTCTGCTAGGGTTG. For whole mtDNA amplification from tissues, 2 fragment PCR reaction was performed employing the following primers, fragment 1: F-3222 GGATCCTACTCTCTACAAAC and R- 11432 TAGTTTGCCGCGTTGGGTGG; fragment 2: F-11271 CTACCCCCTTCAATCAATCT and R- 3335 CCGGTTTGTTTCTGCTAGGG. PCR conditions for single PCR reaction were: one cycle at 94°C for 3 min, followed by 30 cycles at 94°C for 30 sec, 56°C for 1 min and 68 ^o^C for 16 min, and then one cycle at 72°C for 10 min with TAKARA LA Taq kit (Clontech). Two fragment PCR reaction was performed under the following conditions: one cycle at 94°C for 5 min, then 30 cycles at 94°C for 30 sec, 56°C for 20 sec and 68 ^o^C for 7 min, and followed by one cycle at 68°C for 3 min.

### Whole mtDNA sequencing by MiSeq

Whole mtDNA sequencing was performed as described previously [13]. Briefly, the concentration of full length mtDNA PCR products was measured by Qubit 2.0 Fluorometer (Invitrogen) and library preparation was performed using Nextera XT DNA sample preparation kits (Illumina) following the manufacturer’s manual. Sequencing was performed on the Illumina MiSeq platform and the data were analyzed using NextGENe software. Sequence reads ranging from 250–500 bps were quality filtered and processed using NextGENe software and an algorithm similar to BLAT. Sequence error correction feature (condensation) was performed to reduce false positive variants and produce consensus sequence and variant calls for each sample. Alignment without sequence condensation was used to calculate the percentage of the mitochondrial genome with depth of coverage of 1000. Starting from quality FASTQ reads, the reads were filtered for quality and converted to FASTA format. Filtered reads were then aligned to the mouse mitochondrial sequence reference AY172335 followed by variant calling. Variant heteroplasmy was calculated by NextGENe software as follows: Base Heteroplasmy (Mutant Allele Frequency %) = mutant allele (forward + reverse) / total coverage of all alleles C, G, T, A (Forward + Reverse) *100.

### Mitochondrial respiratory chain enzyme activity assays

Mitochondrial-enriched fractions were generated from each iPS or ESC homogenate as described previously [60]. Mitochondrial respiratory chain complex I and IV activities were measured in mitochondrial-enriched samples by spectrophotometric methods [60]. A VersaMax microplate reader (Molecular Devices) in combination with SoftMax Pro software was used for activity measurements and calculations. Complex I activities were expressed as percent relative to rotenone inhibition and complex IV activities were expressed as nmol per min per mg protein.

### Statistical analysis

Student’s t-test was used for all comparisons involved in the study. A P-value less than 0.05 was considered significant. No statistical methods were used to predetermine sample size.

### Accession numbers

The SRA accession number for processed Miseq datasets reported in this paper is SRP099227.

## Supporting information

S1 Table Distribution of mtDNA mutations in wild type mice. (XLSX)

S2 Table Early embryonic mutations in individual SF and iPS clones from wild type mice. (XLSX)

S3 Table Number and heteroplasmy of germline mtDNA mutations in *Polg* mice with aging. (XLSX)

S4 Table Number and heteroplasmy of early embryonic mtDNA mutations in *Polg* mice with aging. (XLSX)

S5 Table MtDNA mutations in *Polg* females and their offspring. (XLSX)

S6 Table List of shared and unique mtDNA mutations in single SF clones in *Polg* mice with aging. (XLSX)

S7 Table List of functional somatic mutations with heteroplasmy ≥15% in *Polg* mice with aging. (XLSX)

## References

[1] Wallace DC. Why do we still have a maternally inherited mitochondrial DNA? Insights from evolutionary medicine. Annual review of biochemistry. 2007;76:781–821. 10.1146/annurev.biochem.76.081205.150955 . 17506638

[2] Anderson S, Bankier AT, Barrell BG, de Bruijn MH, Coulson AR, Drouin J, et al Sequence and organization of the human mitochondrial genome. Nature. 1981;290(5806):457–65. Epub 1981/04/09. . 7219534 10.1038/290457a0

[3] Wallace DC. A mitochondrial bioenergetic etiology of disease. The Journal of clinical investigation. 2013;123(4):1405–12. 10.1172/JCI61398 ; PubMed Central PMCID: PMC3614529. 23543062 PMC3614529

[4] Harman D. Aging: a theory based on free radical and radiation chemistry. Journal of gerontology. 1956;11(3):298–300. Epub 1956/07/01. . 13332224 10.1093/geronj/11.3.298

[5] Trifunovic A, Larsson NG. Mitochondrial dysfunction as a cause of ageing. Journal of internal medicine. 2008;263(2):167–78. Epub 2008/01/30. 10.1111/j.1365-2796.2007.01905.x . 18226094

[6] Kirkwood TB. Understanding the odd science of aging. Cell. 2005;120(4):437–47. Epub 2005/03/01. 10.1016/j.cell.2005.01.027 . 15734677

[7] Szilard L. ON THE NATURE OF THE AGING PROCESS. Proceedings of the National Academy of Sciences of the United States of America. 1959;45(1):30–45. Epub 1959/01/01. ; PubMed Central PMCID: PMCPMC222509. 16590351 10.1073/pnas.45.1.30 PMC222509

[8] Greco M, Villani G, Mazzucchelli F, Bresolin N, Papa S, Attardi G. Marked aging-related decline in efficiency of oxidative phosphorylation in human skin fibroblasts. FASEB journal: official publication of the Federation of American Societies for Experimental Biology. 2003;17(12):1706–8. Epub 2003/09/06. 10.1096/fj.02-1009fje . 12958183

[9] Mishra P, Chan DC. Mitochondrial dynamics and inheritance during cell division, development and disease. Nature reviews Molecular cell biology. 2014;15(10):634–46. 10.1038/nrm3877 ; PubMed Central PMCID: PMCPMC4250044. 25237825 PMC4250044

[10] Corral-Debrinski M, Horton T, Lott MT, Shoffner JM, Beal MF, Wallace DC. Mitochondrial DNA deletions in human brain: regional variability and increase with advanced age. Nature genetics. 1992;2(4):324–9. Epub 1992/12/01. 10.1038/ng1292-324 . 1303288

[11] Herbst A, Pak JW, McKenzie D, Bua E, Bassiouni M, Aiken JM. Accumulation of mitochondrial DNA deletion mutations in aged muscle fibers: evidence for a causal role in muscle fiber loss. The journals of gerontology Series A, Biological sciences and medical sciences. 2007;62(3):235–45. Epub 2007/03/29. ; PubMed Central PMCID: PMCPMC2846622. 17389720 10.1093/gerona/62.3.235 PMC2846622

[12] Dunbar DR, Moonie PA, Swingler RJ, Davidson D, Roberts R, Holt IJ. Maternally transmitted partial direct tandem duplication of mitochondrial DNA associated with diabetes mellitus. Human molecular genetics. 1993;2(10):1619–24. Epub 1993/10/01. . 8268914 10.1093/hmg/2.10.1619

[13] Kang E, Wang X, Tippner-Hedges R, Ma H, Folmes Clifford DL, Gutierrez Nuria M, et al Age-Related Accumulation of Somatic Mitochondrial DNA Mutations in Adult-Derived Human iPSCs. Cell stem cell. 2016 10.1016/j.stem.2016.02.005 27151456

[14] Keogh M, Chinnery PF. Hereditary mtDNA heteroplasmy: a baseline for aging? Cell metabolism. 2013;18(4):463–4. Epub 2013/10/08. 10.1016/j.cmet.2013.09.015 . 24093673

[15] Michikawa Y, Mazzucchelli F, Bresolin N, Scarlato G, Attardi G. Aging-dependent large accumulation of point mutations in the human mtDNA control region for replication. Science. 1999;286(5440):774–9. Epub 1999/10/26. . 10531063 10.1126/science.286.5440.774

[16] Khrapko K, Nekhaeva E, Kraytsberg Y, Kunz W. Clonal expansions of mitochondrial genomes: implications for in vivo mutational spectra. Mutat Res. 2003;522(1–2):13–9. Epub 2003/01/09. . 12517407 10.1016/s0027-5107(02)00306-8

[17] Khrapko K, Ebralidse K, Kraytsberg Y. Where and when do somatic mtDNA mutations occur? Ann N Y Acad Sci. 2004;1019:240–4. Epub 2004/07/13. 10.1196/annals.1297.040 . 15247022

[18] Khrapko K. The timing of mitochondrial DNA mutations in aging. Nature genetics. 2011;43(8):726–7. Epub 2011/07/28. 10.1038/ng.895 ; PubMed Central PMCID: PMCPMC3670970. 21792237 PMC3670970

[19] Nicholas A, Kraytsberg Y, Guo X, Khrapko K. On the timing and the extent of clonal expansion of mtDNA deletions: evidence from single-molecule PCR. Experimental neurology. 2009;218(2):316–9. Epub 2009/05/12. 10.1016/j.expneurol.2009.04.029 ; PubMed Central PMCID: PMCPMC2724894. 19426731 PMC2724894

[20] Taylor SD, Ericson NG, Burton JN, Prolla TA, Silber JR, Shendure J, et al Targeted enrichment and high-resolution digital profiling of mitochondrial DNA deletions in human brain. Aging cell. 2014;13(1):29–38. Epub 2013/08/06. 10.1111/acel.12146 ; PubMed Central PMCID: PMCPMC4068027. 23911137 PMC4068027

[21] Larsson NG. Somatic mitochondrial DNA mutations in mammalian aging. Annual review of biochemistry. 2010;79:683–706. 10.1146/annurev-biochem-060408-093701 . 20350166

[22] Linnane AW, Marzuki S, Ozawa T, Tanaka M. Mitochondrial DNA mutations as an important contributor to ageing and degenerative diseases. Lancet (London, England). 1989;1(8639):642–5. Epub 1989/03/25. . 2564461 10.1016/s0140-6736(89)92145-4

[23] Ross JM, Stewart JB, Hagstrom E, Brene S, Mourier A, Coppotelli G, et al Germline mitochondrial DNA mutations aggravate ageing and can impair brain development. Nature. 2013;501(7467):412–5. Epub 2013/08/24. 10.1038/nature12474 ; PubMed Central PMCID: PMC3820420. 23965628 PMC3820420

[24] Trifunovic A, Wredenberg A, Falkenberg M, Spelbrink JN, Rovio AT, Bruder CE, et al Premature ageing in mice expressing defective mitochondrial DNA polymerase. Nature. 2004;429(6990):417–23. Epub 2004/05/28. 10.1038/nature02517 . 15164064

[25] Kujoth GC, Hiona A, Pugh TD, Someya S, Panzer K, Wohlgemuth SE, et al Mitochondrial DNA mutations, oxidative stress, and apoptosis in mammalian aging. Science. 2005;309(5733):481–4. Epub 2005/07/16. 10.1126/science.1112125 . 16020738

[26] Vermulst M, Bielas JH, Kujoth GC, Ladiges WC, Rabinovitch PS, Prolla TA, et al Mitochondrial point mutations do not limit the natural lifespan of mice. Nature genetics. 2007;39(4):540–3. Epub 2007/03/06. 10.1038/ng1988 . 17334366

[27] Ameur A, Stewart JB, Freyer C, Hagstrom E, Ingman M, Larsson NG, et al Ultra-deep sequencing of mouse mitochondrial DNA: mutational patterns and their origins. PLoS genetics. 2011;7(3):e1002028 Epub 2011/04/02. 10.1371/journal.pgen.1002028 ; PubMed Central PMCID: PMCPMC3063763. 21455489 PMC3063763

[28] Khaidakov M, Heflich RH, Manjanatha MG, Myers MB, Aidoo A. Accumulation of point mutations in mitochondrial DNA of aging mice. Mutat Res. 2003;526(1–2):1–7. Epub 2003/04/26. . 12714177 10.1016/s0027-5107(03)00010-1

[29] Jazin EE, Cavelier L, Eriksson I, Oreland L, Gyllensten U. Human brain contains high levels of heteroplasmy in the noncoding regions of mitochondrial DNA. Proceedings of the National Academy of Sciences of the United States of America. 1996;93(22):12382–7. Epub 1996/10/29. ; PubMed Central PMCID: PMCPMC38000. 8901590 10.1073/pnas.93.22.12382 PMC38000

[30] Chinnery PF, Turnbull DM. Mitochondrial DNA mutations in the pathogenesis of human disease. Molecular medicine today. 2000;6(11):425–32. Epub 2000/11/14. . 11074368 10.1016/s1357-4310(00)01805-0

[31] Wallace DC, Chalkia D. Mitochondrial DNA genetics and the heteroplasmy conundrum in evolution and disease. Cold Spring Harbor perspectives in biology. 2013;5(11):a021220 10.1101/cshperspect.a021220 . 24186072 PMC3809581

[32] Hamalainen RH, Manninen T, Koivumaki H, Kislin M, Otonkoski T, Suomalainen A. Tissue- and cell-type-specific manifestations of heteroplasmic mtDNA 3243A>G mutation in human induced pluripotent stem cell-derived disease model. Proceedings of the National Academy of Sciences of the United States of America. 2013;110(38):E3622–30. 10.1073/pnas.1311660110 ; PubMed Central PMCID: PMC3780874. 24003133 PMC3780874

[33] Muller FL, Lustgarten MS, Jang Y, Richardson A, Van Remmen H. Trends in oxidative aging theories. Free Radic Biol Med. 2007;43(4):477–503. 10.1016/j.freeradbiomed.2007.03.034 . 17640558

[34] Cheng KC, Cahill DS, Kasai H, Nishimura S, Loeb LA. 8-Hydroxyguanine, an abundant form of oxidative DNA damage, causes G—-T and A—-C substitutions. The Journal of biological chemistry. 1992;267(1):166–72. . 1730583

[35] Kennedy SR, Salk JJ, Schmitt MW, Loeb LA. Ultra-sensitive sequencing reveals an age-related increase in somatic mitochondrial mutations that are inconsistent with oxidative damage. PLoS genetics. 2013;9(9):e1003794 Epub 2013/10/03. 10.1371/journal.pgen.1003794 ; PubMed Central PMCID: PMCPMC3784509. 24086148 PMC3784509

[36] Williams SL, Mash DC, Zuchner S, Moraes CT. Somatic mtDNA mutation spectra in the aging human putamen. PLoS genetics. 2013;9(12):e1003990 Epub 2013/12/18. 10.1371/journal.pgen.1003990 ; PubMed Central PMCID: PMCPMC3854840. 24339796 PMC3854840

[37] DiMauro S, Schon EA. Mitochondrial respiratory-chain diseases. The New England journal of medicine. 2003;348(26):2656–68. Epub 2003/06/27. 10.1056/NEJMra022567 . 12826641

[38] Balaban RS, Nemoto S, Finkel T. Mitochondria, oxidants, and aging. Cell. 2005;120(4):483–95. Epub 2005/03/01. 10.1016/j.cell.2005.02.001 . 15734681

[39] Sbisa E, Tanzariello F, Reyes A, Pesole G, Saccone C. Mammalian mitochondrial D-loop region structural analysis: identification of new conserved sequences and their functional and evolutionary implications. Gene. 1997;205(1–2):125–40. Epub 1998/02/14. . 9461386 10.1016/s0378-1119(97)00404-6

[40] Uhler JP, Falkenberg M. Primer removal during mammalian mitochondrial DNA replication. DNA Repair (Amst). 2015;34:28–38. 10.1016/j.dnarep.2015.07.003 . 26303841

[41] McInerny SC, Brown AL, Smith DW. Region-specific changes in mitochondrial D-loop in aged rat CNS. Mechanisms of ageing and development. 2009;130(5):343–9. Epub 2009/05/12. 10.1016/j.mad.2009.01.008 . 19428453

[42] Trifunovic A, Hansson A, Wredenberg A, Rovio AT, Dufour E, Khvorostov I, et al Somatic mtDNA mutations cause aging phenotypes without affecting reactive oxygen species production. Proceedings of the National Academy of Sciences of the United States of America. 2005;102(50):17993–8. Epub 2005/12/08. 10.1073/pnas.0508886102 ; PubMed Central PMCID: PMCPMC1312403. 16332961 PMC1312403

[43] Falkenberg M, Larsson NG, Gustafsson CM. DNA replication and transcription in mammalian mitochondria. Annual review of biochemistry. 2007;76:679–99. Epub 2007/04/06. 10.1146/annurev.biochem.76.060305.152028 . 17408359

[44] Wallace DC, Fan W. Energetics, epigenetics, mitochondrial genetics. Mitochondrion. 2010;10(1):12–31. 10.1016/j.mito.2009.09.006 ; PubMed Central PMCID: PMC3245717. 19796712 PMC3245717

[45] Lanza IR, Nair KS. Mitochondrial function as a determinant of life span. Pflugers Archiv: European journal of physiology. 2010;459(2):277–89. Epub 2009/09/17. 10.1007/s00424-009-0724-5 ; PubMed Central PMCID: PMCPMC2801852. 19756719 PMC2801852

[46] Gomes AP, Price NL, Ling AJ, Moslehi JJ, Montgomery MK, Rajman L, et al Declining NAD(+) induces a pseudohypoxic state disrupting nuclear-mitochondrial communication during aging. Cell. 2013;155(7):1624–38. Epub 2013/12/24. 10.1016/j.cell.2013.11.037 ; PubMed Central PMCID: PMCPMC4076149. 24360282 PMC4076149

[47] Cerutti R, Pirinen E, Lamperti C, Marchet S, Sauve AA, Li W, et al NAD(+)-dependent activation of Sirt1 corrects the phenotype in a mouse model of mitochondrial disease. Cell metabolism. 2014;19(6):1042–9. Epub 2014/05/13. 10.1016/j.cmet.2014.04.001 ; PubMed Central PMCID: PMCPMC4051987. 24814483 PMC4051987

[48] Edgar D, Shabalina I, Camara Y, Wredenberg A, Calvaruso MA, Nijtmans L, et al Random point mutations with major effects on protein-coding genes are the driving force behind premature aging in mtDNA mutator mice. Cell metabolism. 2009;10(2):131–8. Epub 2009/08/07. 10.1016/j.cmet.2009.06.010 . 19656491

[49] He Y, Wu J, Dressman DC, Iacobuzio-Donahue C, Markowitz SD, Velculescu VE, et al Heteroplasmic mitochondrial DNA mutations in normal and tumour cells. Nature. 2010;464(7288):610–4. Epub 2010/03/05. 10.1038/nature08802 ; PubMed Central PMCID: PMCPMC3176451. 20200521 PMC3176451

[50] Vermulst M, Wanagat J, Kujoth GC, Bielas JH, Rabinovitch PS, Prolla TA, et al DNA deletions and clonal mutations drive premature aging in mitochondrial mutator mice. Nature genetics. 2008;40(4):392–4. 10.1038/ng.95 . 18311139

[51] Li M, Schonberg A, Schaefer M, Schroeder R, Nasidze I, Stoneking M. Detecting heteroplasmy from high-throughput sequencing of complete human mitochondrial DNA genomes. American journal of human genetics. 2010;87(2):237–49. Epub 2010/08/11. 10.1016/j.ajhg.2010.07.014 ; PubMed Central PMCID: PMCPMC2917713. 20696290 PMC2917713

[52] Payne BA, Wilson IJ, Yu-Wai-Man P, Coxhead J, Deehan D, Horvath R, et al Universal heteroplasmy of human mitochondrial DNA. Human molecular genetics. 2013;22(2):384–90. Epub 2012/10/19. 10.1093/hmg/dds435 ; PubMed Central PMCID: PMCPMC3526165. 23077218 PMC3526165

[53] Popadin K, Safdar A, Kraytsberg Y, Khrapko K. When man got his mtDNA deletions? Aging cell. 2014;13(4):579–82. Epub 2014/06/05. 10.1111/acel.12231 ; PubMed Central PMCID: PMCPMC4326951. 24894296 PMC4326951

[54] Greaves LC, Nooteboom M, Elson JL, Tuppen HA, Taylor GA, Commane DM, et al Clonal expansion of early to mid-life mitochondrial DNA point mutations drives mitochondrial dysfunction during human ageing. PLoS genetics. 2014;10(9):e1004620 Epub 2014/09/19. 10.1371/journal.pgen.1004620 ; PubMed Central PMCID: PMCPMC4169240. 25232829 PMC4169240

[55] Elson JL, Samuels DC, Turnbull DM, Chinnery PF. Random intracellular drift explains the clonal expansion of mitochondrial DNA mutations with age. American journal of human genetics. 2001;68(3):802–6. Epub 2001/02/17. 10.1086/318801 ; PubMed Central PMCID: PMCPMC1274494. 11179029 PMC1274494

[56] Payne BA, Wilson IJ, Hateley CA, Horvath R, Santibanez-Koref M, Samuels DC, et al Mitochondrial aging is accelerated by anti-retroviral therapy through the clonal expansion of mtDNA mutations. Nature genetics. 2011;43(8):806–10. Epub 2011/06/28. 10.1038/ng.863 ; PubMed Central PMCID: PMCPMC3223397. 21706004 PMC3223397

[57] Greaves LC, Barron MJ, Campbell-Shiel G, Kirkwood TB, Turnbull DM. Differences in the accumulation of mitochondrial defects with age in mice and humans. Mechanisms of ageing and development. 2011;132(11–12):588–91. Epub 2011/10/22. 10.1016/j.mad.2011.10.004 . 22015485

[58] Guo X, Kudryavtseva E, Bodyak N, Nicholas A, Dombrovsky I, Yang D, et al Mitochondrial DNA deletions in mice in men: substantia nigra is much less affected in the mouse. Biochimica et biophysica acta. 2010;1797(6–7):1159–62. Epub 2010/04/15. 10.1016/j.bbabio.2010.04.005 ; PubMed Central PMCID: PMCPMC2891141. 20388490 PMC2891141

[59] Morris J, Na YJ, Zhu H, Lee JH, Giang H, Ulyanova AV, et al Pervasive within-Mitochondrion Single-Nucleotide Variant Heteroplasmy as Revealed by Single-Mitochondrion Sequencing. Cell reports. 2017;21(10):2706–13. Epub 2017/12/07. 10.1016/j.celrep.2017.11.031 ; PubMed Central PMCID: PMCPMC5771502. 29212019 PMC5771502

[60] Spinazzi M, Casarin A, Pertegato V, Salviati L, Angelini C. Assessment of mitochondrial respiratory chain enzymatic activities on tissues and cultured cells. Nature protocols. 2012;7(6):1235–46. 10.1038/nprot.2012.058 . 22653162

